# Novel *Erwinia persicina* Infecting Phage Midgardsormr38 Within the Context of Temperate *Erwinia* Phages

**DOI:** 10.3389/fmicb.2020.01245

**Published:** 2020-06-19

**Authors:** Nikita Zrelovs, Andris Dislers, Andris Kazaks

**Affiliations:** Latvian Biomedical Research and Study Centre, Riga, Latvia

**Keywords:** bacteriophage, *Erwinia*, prophage, lysogeny, comparative genomics, complete genome, evolutionary relationships, bioinformatics

## Abstract

Prophages or prophage remnants are found in chromosomes of many bacterial strains and might increase the environmental fitness and/or virulence of their hosts. Up to this date, complete genome sequences of only seven temperate bacteriophages infecting bacteria from genus *Erwinia*, comprising of mostly phytopathogenic bacteria, are available publicly. No attempts to analyze the global diversity of temperate *Erwinia* phages and establish relationships between cultured temperate *Erwinia* phages and prophages were yet made. In this study, we have isolated, sequenced, and described novel *Erwinia persicina* infecting bacteriophage “Midgardsormr38” and placed it in the context of previously described *Erwinia* sp. temperate phages and putative prophages derived from chromosomes of publicly available complete genomes of *Erwinia* sp. to broaden and investigate diversity of temperate *Erwinia* phages based on their genomic contents. The study revealed more than 50 prophage or prophage remnant regions in the genomes of different *Erwinia* species. At least 5 of them seemed to be intact and might represent novel inducible *Erwinia* phages. Given the enormous bacteriophage diversity, attempts to establish evolutionary relationships between temperate *Erwinia* phages revealed at least five different clusters of temperate phages sharing higher degree of similarity.

## Introduction

Bacteriophages, shortly phages—viruses of bacteria, the most abundant biological entities known to man—are omnipresent in every natural habitat where bacteria thrive (Clokie et al., 2011). Despite the fact that we have yet observed and described only a minor fraction of the existing phage diversity, phages are believed to harbor the most diverse genetic pool, with millions of different phage species expected to exist, and have already proven themselves as a treasury of valuable genes, products of which not only have notably advanced the development of molecular biology but also have given raise to various phage or phage protein derived practical applications (Schoenfeld et al., 2010; Salmond and Fineran, 2015; Hudson et al., 2016). Today’s alarming state of antibiotic resistance spread throughout the kingdom Bacteria has reinvigorated interest in bacteriophages, mainly as natural biocontrol agents to fight bacterial infections (Drulis-Kawa et al., 2015).

Bacteriophages can be classified by numerous characteristics, such as possible life cycles, nucleic acid contents, and virion morphology, among many others. The majority of the so far observed phages belong to the viral order *Caudovirales*, comprising tailed double-stranded DNA containing bacteriophages (Ackermann, 2009). Based on their life cycle, most of the bacteriophages can be divided into two major groups, lytic and temperate phages. Successful infection of the susceptible host by lytic (virulent) bacteriophage almost certainly results in lysis of the host cell and release of phage progeny capable of further infections into the surrounding environment (Young, 1992), whereas infection by a lysogenic (temperate) phage might result in incorporation of phage nucleic acid into the genome of its host as either part of its chromosome or in the form of an episome (Utter et al., 2014; Howard-Varona et al., 2017). Once integrated in the host’s genome, temperate bacteriophages enter the dormant state suppressing transcription from most of their genes and becoming a prophage and making bacteria, a so-called lysogen, sometimes providing particular environmental fitness or virulence to its host (Davies et al., 2016; Harrison and Brockhurst, 2017; Howard-Varona et al., 2017). Prophages residing in genomes of bacteria, however, can be induced to enter the lytic pathway leading to bacterial lysis and release of progeny (Mackey et al., 2016). Despite the possibility of engineering virulent derivatives of temperate phages incapable of lysogenizing their host, considerably larger efforts were and are being made to explore the diversity of naturally lytic phages due to easier possibility of their use in biocontrol, leaving temperate phages of many bacterial taxa in their shadow. Nevertheless, thorough characterization and classification of every newly discovered bacteriophage, regardless of its life cycle, is of utter importance for understanding the prospects and limitations of its possible use (Hyman, 2019).

Genus *Erwinia* is known to include Gram-negative, mostly phytopathogenic, bacteria that mainly cause blights, wilts, and soft rot in various plants (Brenner et al., 2005). Pathogenic *Erwinia* usually infect the susceptible plant through natural openings and wounds, starting to cause damage in the vascular tissue before spreading further throughout the plant. The economic losses attributed to just the type species *Erwinia amylovora* that causes fire blight symptoms in numerous *Rosaceae* (e.g., pears and apples) plants are estimated to exceed hundreds of millions United States dollars annually in the United States alone (Norelli et al., 2003). Soft rot, fleshy crop targeting bacterial disease with outbreak possibility during all the stages of crop production—in the field, during transit and storage, and even during marketing—is thought to be primarily caused by bacteria from genus *Erwinia* as well (Charkowski, 2006; Bhat et al., 2010). Strains of *E. persicina*, the host of novel *Erwinia*-infecting bacteriophage Midgardsormr38, in addition to insect gut as in present study, were previously isolated not only from plant sources such as lucerne, soybean, garlic, onions, common bean, pea, cucumber, tomato, melon, apple, and pear but also from human urinary tract and even biofilms from paleolithic rock paintings (Hao et al., 1990; O’Hara et al., 1998; Kiessling et al., 2005; Zhang and Nan, 2014). Although not common, some of the *Erwinia* species (e.g., *Erwinia billingae*, *E. persicina*, and *Erwinia tasmaniensis*-like organism) were found to be associated with disease in humans (O’Hara et al., 1998; Shin et al., 2008; Prod’homme et al., 2017).

Current common methods for control of pathogenic *Erwinia* sp. include the use of bactericidal copper compounds, antibiotic treatment of plants, removal of the diseased tissue, and even substantial planted areas to prevent the spread of the infection, plant crop varieties less prone to infection, and use antagonistic bacteria—a detailed review of these methods is out of the scope of this paper and has been discussed elsewhere previously (Norelli et al., 2003; Sundin et al., 2016; Ait Bahadou et al., 2018; Lamichhane et al., 2018). An alternative approach that begins to draw more and more attention and has a proven history of use as an alternative means of pathogenic bacteria biocontrol is usage of bacteriophages to naturally diminish the number of bacterial pathogens in the course of continuously multiplying infection upon contact with the susceptible cells (Buttimer et al., 2017). Despite the fact that lytic phages obviously fit the role of such a biocontrol agent by a considerably greater margin than the temperate ones, recently, a study successfully employing lytic derivatives of broad host range temperate *Mycobacterium* phages in a phage therapy cocktail for treatment of *Mycobacterium abscessus* infection has been published (Dedrick et al., 2019a,b).

Currently, there are about 60 phages known to infect *Erwinia* sp., a vast majority of them being lytic *E. amylovora* infecting viruses. Only seven temperate *Erwinia* phage sequences are publicly available up to date. Here we report characterization and complete genome sequence of novel temperate *E. persicina* infecting phage Midgardsormr38 and try to broaden our knowledge on the diversity and evolutionary relationships of cultured and putative temperate *Erwinia* bacteriophages, which were largely ignored in the literature, apart from a few exceptions, either in papers describing novel temperate *Erwinia* phages or as an accessory part of a broader phage group analyses (Grose and Casjens, 2014; Sharma et al., 2019; Thompson et al., 2019).

## Materials and Methods

### Host Selection

About 1 g of locally collected dead fruit flies (*Drosophila melanogaster*) was melted in a pestle, suspended in 10 mL of physiological saline and allowed to settle down at +4°C overnight. 5–50 μL aliquots of supernatant were plated on agarized (1.5%) lysogeny broth (LB): 10 g/L peptone from casein, enzymatic digest (Sigma-Aldrich), 5 g/L yeast extract (Fluka), 10 g/L NaCl, and 15 g/L granulated agar (Difco), prepared in distilled water, with further incubation at room temperature (RT) for 3 days. Individual colonies of morphologically different types (about 10) were picked up and purified by subculturing and suspended in 5 mL of LB, incubated stationary at RT to obtain the indicator cultures.

### Phage Selection

Phage Midgardsormr38 was selected from the suspension of about 2 g of locally collected mixed dead insects: houseflies (*Musca domestica*), lady beetles (*Coccinellidae*), and green lacewings (*Chrysopidae*) collected and processed as in the case of host selection. The supernatant was then centrifuged on a bench-top centrifuge at RT (18,400 × *g*) for 30 min and filtered through a 0.45-μm pore size filter (Sarstedt).

Phage isolation was performed using the traditional double-agar overlay plaque-assay technique as described elsewhere (Gratia, 1936). Briefly, aliquots (5 and 50 μL) of the phage-containing filtered supernatant were mixed with 100 μL of previously obtained indicator cultures and 7 mL of soft LB agar (0.7%) and plated onto the agarized (1.5%) LB as a bottom layer; plates were incubated overnight at +30°C. In the case of an indicator culture named “Dr4,” about 50 plaques were observed on a plate with 50 μL of supernatant used. An individual plaque was picked up, purified by subculturing, and transferred onto a fresh plate with an indicator culture and soft agar followed by incubation overnight at +30°C. Several spots of lysed bacteria from the top agar were collected in the 1.5-mL Eppendorf tube, vortexed well, and centrifuged at RT for 30 min (18,400 × *g*). 5-μL aliquots of the obtained supernatant were then used to achieve near complete lysis of the host lawn on 3 plates. After incubation, the phage was extracted from the top agar layer by intensive vortexing of the collected top agar material in a 50-mL Falcon tube with an addition of physiological saline (20% by the volume of solid agar material), centrifugation (RT, 30 min, 12,500 × *g*), and filtration of the supernatant (0.45 μm filter). The phage titer obtained was around 10^10^ pfu/mL. For the next step of phage scale-up propagation, the host bacteria were cultivated in a 1-L flask with 200 mL of liquid LB medium at +30°C with aeration (200 rpm) until OD_540_ reached 0.3–0.4, followed by inoculation with the phage at a multiplicity of infection (MOI) ∼1.0. Visible lysis occurred the next day, with the phage titer reaching ∼1–2 × 10^10^ pfu/mL. The lysate was then clarified by the centrifugation and filtration as mentioned previously. Phage particles were sedimented at 70,000 × *g* for 1 h in a JA-30.50 Ti rotor (Beckman Coulter). The phage pellet was resuspended in 4 mL of supernatant, and 2-mL aliquots were layered on the top of CsCl solution (0.65 g/mL CsCl in 20 mM Tris–HCl, pH 7.2) in Ultra-Clear centrifuge tubes (14 mm × 95 mm, Beckman Coulter). The samples were then centrifuged at 100,000 × *g* for 20 h in an SW 40 Ti rotor (Beckman Coulter), and the phage-containing band was collected and transferred to phosphate-buffered saline using Illustra NAP-25 columns (GE Healthcare). Phage DNA was then extracted by treating the resulting sample with 0.5% SDS and proteinase K (50 μg per mL, Thermo Fisher Scientific) at 56°C for 1 h, followed by standard phenol–chloroform extraction, ethanol precipitation, and resuspension of the phage DNA in dH_2_O (Green and Sambrook, 2017).

### Identification of Host Bacteria

For the taxonomic identification of the Midgardsormr38 host, DNA from an overnight culture acquired from a single phage-susceptible bacterial colony was obtained using MagaZorb^®^ DNA Mini-Prep (Promega) in accordance with the manufacturer’s instructions. Classical PCR using 27F and 1492R primers followed by native agarose gel electrophoresis was performed (Weisburg et al., 1991; Janda and Abbott, 2007).

The band containing a product of approximately 1,450 bp in length was extracted using GeneJET Gel Extraction kit (Thermo Fisher Scientific). 16s rRNA gene sequencing of the purified product was performed using the same aforementioned primers according to the BigDye^TM^ Terminator v3.1 Cycle Sequencing Kit (Thermo Fisher Scientific) protocol. The acquired partial sequence of the 16s rRNA gene was further compared to the 16s rRNA BLAST, EzBioCloud, and RDB Project databases (Altschul et al., 1990; Cole et al., 2014; Yoon et al., 2017). It was revealed that the Midgardsormr38 susceptible bacterial isolate belongs to the genus *Erwinia* and is a strain of *E. persicina*, sharing only minor nucleotide differences with *E. persicina*-type strain NBRC 102418 (GenBank: BCTN01000053).

### Phage Electron-Microscopic Examination

**FIGURE 1:**
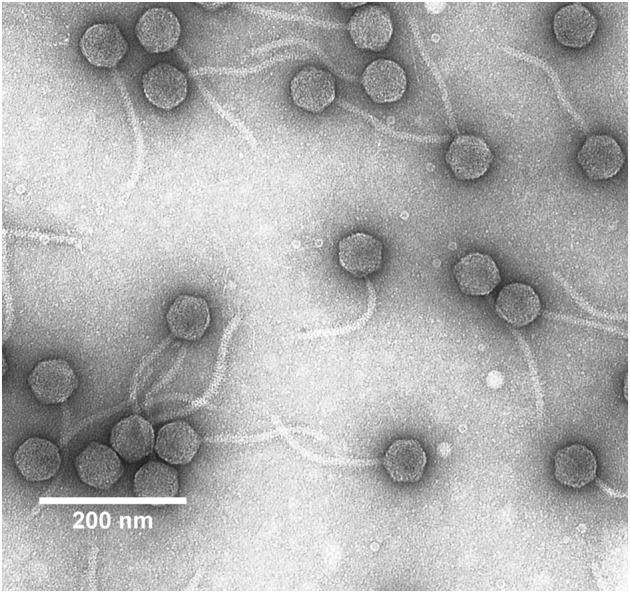
Transmission electron micrograph of *Erwinia persicina* bacteriophage Midgardsormr38 stained with 0.5% uranyl acetate.

Five microliter of the diluted Midgardsormr38 phage stock (∼5 × 10^10^ pfu/mL) were fixed on a Formvar/carbon-coated copper mesh grid for 5 min and then negatively stained using 0.5% uranyl acetate. The phage sample was examined on a JEOL JEM 1230 transmission electron microscope (Figure 1). Electron micrographs were taken using a Morada 11 MegaPixel TEM CCD microscope-mounted camera (Olympus), and phage particle dimensions were determined from a single best micrograph obtained that had numerous intact particles in the field of view using ImageJ v1.52a software (Schneider et al., 2012) with a scale bar as a reference for the pixel to nm ratio. Head diameters and tail lengths of 10 randomly selected phage particles were measured using either straight line (for head diameters) or segmented line (for tail lengths) utilities provided within the software.

### Whole-Genome Sequencing

As a first step in the preparation of the fragment library for WGS, phage DNA was randomly physically sheared using a Covaris S220 focused-ultrasonicator. The barcoded DNA fragment library with the target insert size of 250 bp was prepared using Ion Xpress^TM^ Plus Fragment Library Kit (Thermo Fisher Scientific) following the manufacturer’s guidelines with NucleoMag^®^ NGS Clean-up and Size Select (MACHEREY-NAGEL) bead cleanups in between the library preparation steps. Library concentrations were verified on Agilent 2100 Bioanalyzer using hsDNA assay. Sequencing of the resulting library was carried out on an Ion Proton using Ion PI^TM^ chip (Thermo Fisher Scientific).

### *De novo* Assembly

The sequencing run yielded a total of 108,457 raw reads. After manual quality assessment of the raw data using FastQC tool (Andrews, 2010) for the visualization and statistics, reads were preprocessed by quality trimming using Trimmomatic (Bolger et al., 2014) to improve accuracy of the further assembly. *De novo* assembly was carried out using Mira 4 assembler (Chevreux et al., 1999) and resulted in contigs that were manually scaffolded to represent the draft genome. Raw reads were mapped unto the resultant draft genome, and the sequence alignment map was visualized in the IGV viewer (Robinson et al., 2012) to manually determine the low-coverage regions and possible sites of misassembly; 95.74% could be aligned unto the resultant *de novo* assembled complete genome, providing the average whole-genome coverage of 456×. Ambiguous regions were resolved using Sanger sequencing-based primer walking with custom primers designed to be complementary to the sequences upstream of the regions of interest. Genome terminal redundancy was verified by Sanger-based sequencing (Sanger et al., 1977) with primers designed to reach the putative genome ends. Genome termini sequencing was performed on genomic phage DNA with custom primers (5′-CATCTTCTGCCAGTGGAAAG-3′ and 5′-CTCAAGGTTCTTCACCTTGTC-3′) ordered at Metabion using BigDye^®^ Terminator v3.1 Cycle Sequencing Kit (Applied Biosystems) according to the manufacturer’s recommendations. The packaging strategy was inferred using sequence alignment map inspection, PhageTerm, and the TerL homology-based approach (Merrill et al., 2016; Zhang et al., 2018).

### Functional Annotation of the Genome

Open reading frames (ORFs) of novel phage Midgardsormr38 were predicted using Glimmer (Delcher et al., 2007), GeneMark (Besemer and Borodovsky, 2005), and Prodigal (Hyatt et al., 2010). The nucleotide sequence from each of the divergent calls from any of the aforementioned software was translated into the respective amino acid sequence and compared to the non-redundant RefSeq (Pruitt et al., 2007) protein database by homology searches using BLASTp (Altschul et al., 1990). The starting position of any predicted ORF was verified by combining homology search results and manual inspection of the ribosomal binding site (Shine-Dalgarno sequence motif) occurrence upstream of the start codon. Functions of the predicted ORFs were assigned combining convincing BLASTp search results of the products (*E*-value < 1e-10) and HHpred (Söding et al., 2005) where appropriate. The genome was scanned for tRNA sequences using Aragorn (Laslett and Canback, 2004) and tRNA scan (Lowe and Eddy, 1997) software.

### Identification of Putative Prophage Regions in Publicly Available Complete Genomes of *Erwinia* sp.

Accession numbers of complete genomes from *Erwinia* sp. were retrieved from the Nucleotide database using the following search result filter: {Complete genome[All Fields] AND (“Erwinia”[Organism] AND bacteria[filter])} AND {bacteria[filter] AND biomol_genomic[PROP] AND ddbj_embl_genbank[filter] AND (“1000000”[SLEN]: “10000000”[SLEN])} (Clark et al., 2016).

Accession numbers of the selected genomes were then separately used as an input for PHASTER to identify possible prophage regions and to evaluate their completeness (Zhou et al., 2011; Arndt et al., 2016).

The nucleotide sequence of each putative prophage region assumed to be “intact” by PHASTER was manually retrieved to local storage in the form of a fasta file. Retrieved prophage sequences were named after the respective host species/strain of the particular prophage as a basename with an addition of the region number from PHASTER prediction for further unambiguous identification.

The Prophage/Virus database used by PHASTER was downloaded locally, and sequences either entitled “hypothetical protein” or containing placeholders, such as “ORF” and “gp” followed by a number in the title, were removed using awk (Aho et al., 1979).

Prokka was then used for automated genome annotations by gene product homology with the aforementioned database serving as the 1st-priority annotation source using a lowered similarity *e*-value cutoff of 1e-03 and minimum query coverage on query protein of 30% for hits (Seemann, 2014).

### Gene Product Functional Grouping and Color Coding

GenBank file CDS features of both Prokka-generated possible prophage region annotations and publicly available temperate *Erwinia* phage complete genome annotations by the authors of the original submissions were then manually color coded based on their product’s putative function for further visualizations with addition of the “/color = ” qualifier for each CDS. Color coding (RGB) for gene product functional groups retained throughout this paper is as follows: yellow (255 255 0)—proteins involved in virion morphogenesis; red (255 0 0)—regulatory proteins; blue (0 0 255)—replication, modification and repair; green (0 255 0)—lysis; cyan (0 255 255)—lysogeny; purple (178 58 238)—additional functions; and gray (100 100 100)—unknown function.

### Fragmented Alignment of Temperate *Erwinia* Phage Genomes

Genomes of both cultured and predicted temperate phages of *Erwinia* sp., as well as that of an “outgroup”—*Bdellovibrio* phage phi1422—were subjected to fragmented genome alignment using “accurate” BLASTn parameters in Gegenees (fragment size: 200, sliding step size: 100) (Ågren et al., 2012). The cutoff threshold for non-conserved material was set to 5% (the data were normalized against the maximum score that could be obtained with the fragment, and fragments falling under the threshold were not used to calculate the average similarity value), and the resulting autosorted heatmap was exported in the form of NJ tree (Saitou and Nei, 1987) in Nexus format, which was then visualized using SplitsTree4 to generate the phylogram (Huson, 1998).

Related temperate phage and prophage annotated complete genome GenBank files with each gene product color coded were then used for pairwise nucleotide sequence comparisons carried out in Easyfig (Sullivan et al., 2011).

### Shared Gene Content Analysis

Each cluster of phages was subjected to shared gene content analysis using Roary with minimum percentage identity for BLASTp set to 50%. Prokka generated ^∗^.gff files were used as an input in case of derived prophages, while cultured phage complete genome ^∗^.gb files retrieved from the NCBI nucleotide database were first turned into ^∗^.gff file format using Bio:Perl script bp_genbank2gff3.pl as suggested by Roary authors (Page et al., 2015).

The resulting gene presence/absence table was manually modified, redundant columns were removed, and gene loci were colored according to the putative function of their products using the aforementioned color code for each genome separately. Annotation includes all the possible variants of homologous gene annotation from the analyzed genomes and is colored accordingly to the function in majority of the genomes where the homolog is present, whereas, in the case of the two-gene group comprising a hypothetical protein and a protein with putative function assigned, “hypothetical protein” annotation coloring was favored, as was also in the case of ambiguous homolog groups (e.g., same amount of genes of different functional annotations).

### Packaging Strategy Prediction

Terminase large subunit (TerL) or terminase ATPase subunit protein amino acid sequences were retrieved from GenBank complete genome sequence annotation files, publicly available for cultured temperate *Erwinia* phages or Prokka-generated for predicted *Erwinia* prophages based on gene product putative functional predictions. The set of TerL amino acid sequences from phages with an experimentally verified packaging strategy used previously by Merrill et al. (2016) was used to build a packaging strategy prediction inference tree based on TerL sequence homology of different packaging-type terminase large subunits. Multiple amino acid sequence alignment of TerL proteins was performed using Clustal (Thompson et al., 1994), and a neighbor-joining tree was drawn in MEGA (Kumar et al., 2016).

## Results and Discussion

### Morphology of Midgardsormr38

Intact particles of the observed phage had a non-contractile tail of 170.0 ± 2.8 nm length attached to an icosahedral head with the diameter of 59.4 ± 2.0 nm. Combination of morphological features phage Midgardsormr38 exhibited was indicative of it belonging to viral order *Caudovirales*, family *Siphoviridae*.

### Overview of Midgardsormr38 Genome

As was revealed by the uniformity of read coverage during the inspection of the sequence alignment map and inferred from TerL homology-based packaging strategy prediction, *E. persicina* phage Midgardsormr38 employs a headful packaging strategy, resulting in circularly permuted genomes with terminal redundancy of ∼0.4% non-redundant genome length that is unique to each virion. Thus, the non-redundant complete genome of phage Midgardsormr38 is a 50,485-bp-long double-stranded DNA molecule with 50.86% GC content that accommodates a total of 93 predicted ORFs without any identifiable tRNA sequences.

The complete genome nucleotide sequence of phage Midgardsormr38, when aligned to the existing viral entries (taxid: 10239) in the GenBank using BLASTn, shows the highest degree of nucleotide sequence similarity to temperate *Erwinia* phage vB_EhrS_49 (29% query coverage of 69.06% identity). However, when searched among all the taxa, Midgardsormr38, unsurprisingly due to its temperate nature, reveals a comparable or even higher amount of similarity to chromosome regions of species from different bacterial genera (e.g., *Erwinia*, *Enterobacter*, *Salmonella*, and *Citrobacter*, among others).

**FIGURE 2:**
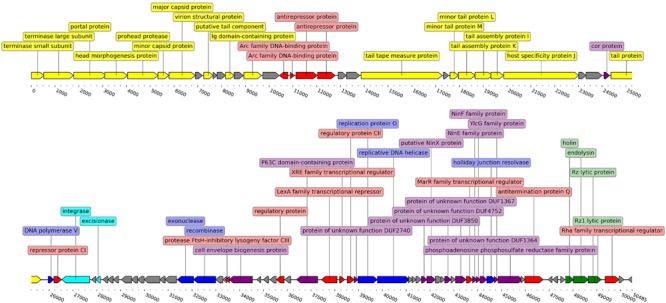
Linearized genome map of phage Midgardsormr38. Black line represents the genome itself. Arrows represent ORFs and show the direction of transcription. Colors are indicative of the predicted functions of respective ORF gene products. Yellow color was assigned to ORFs responsible for phage particle morphogenesis, green—lysis, red—transcription, blue—DNA replication, modification and repair, cyan—lysogeny, purple—additional functions, gray—ORFs encoding proteins with functions unknown. Labels show the putative functions of gene products. The genome map was created using the DNA features viewer (Zulkower and Rosser, 2020).

It was found that the phage genome has a coding capacity of 94.17%, encompassing 55 ORFs with putative functions assigned by homology searches and 38 ORFs for which the predicted function could not be reliably inferred. Three possible phage start codons were found in the genome, 86 ORFs start with ATG, 3 with TTG, and 4 ORFs use CTG as start codon. Sixty-four ORFs are transcribed from a forward strand versus 29 from the reverse strand (Figure 2). Detailed annotation of each ORF from genome of Midgardsormr38 is available in Supplementary Table S1.

### Assessment of Lysogeny

The cells of secondary growth inside the plaques (appearing after 2–3 days of incubation) of plates of phage Midgardsormr38 were spread on an agarized LB plate to obtain individual colonies. After subculturing the resulting individual colonies twice, suspensions of 10 individual colonies were used for phage susceptibility test by the standard double-agar overlay method used for phage titration. Each of the 10 colonies tested was not sensitive to infection by phage Midgardsormr38 when used as a bacterial lawn in double-agar overlay plaque assays. Further analyses, however, showed that lysogenic cells are capable of spontaneous loss of their lysogenic state—uninduced and induced (by UV irradiation) lysogenic cultures both contained infectious centers (phage producing cells) but in different proportion to the total viable cell count.

To induce Midgardsormr38 from the lysogens of the host, three individual phage-resistant colony cell cultures (5 mL with ∼10^8^ CFU/mL) were irradiated in a slowly shaking open Petri dish under TUV 36W SLV/6 (PHILIPS) laboratory germicidal UV lamp (254 nm) for 5 min at 1 m distance (cell viability after irradiation ∼50%). These UV-irradiated cells were then tested for the presence of induced phage by applying 5-μL drops of UV-treated cells unto the layer of control cells (non-lysogenic); all three of irradiated cultures gave clear lytic areas around the edges of the spot of transfer. However, when uninduced cells were tested in the same way, all of the ten prophage-containing cultures produced a smaller turbid lysis zone around the spot of transfer. From these observations, the conclusion that self-induction takes place in the case of prophage Midgardsormr38-containing lysogens and that Midgardsormr38 prophage state is unstable has been made.

The temperate nature of the phage Midgardsormr38 is further supported by the functional annotation of ORF35, encoding a putative integrase highly homologous to the integrases found in other temperate *Erwinia* phages, as well as by numerous phage Lambda lysogeny-related regulatory gene product homologs (cI repressor, cII activator, protease inhibitor cIII) present in the genome of Midgardsormr38.

### Prophage Prediction in the Genomes of *Erwinia* sp.

Nine complete genome sequences of species within the genus *Erwinia* available at the time of writing were retrieved from GenBank and analyzed for the presence of putative prophage regions using PHASTER (Table 1). None of the genomes was free from prophage-like genetic material, although three of the genomes contained no intact prophages, as predicted by PHASTER. Six of the analyzed strains contained at least one prophage region assumed to be intact, while one of them, *Erwinia tracheiphila* strain MDcuke (CP013970.1), revealed a whopping number of 28 putative prophage regions spanning across approximately 17.7% of host’s chromosome length, 19 of which were considered “intact” by PHASTER. Although the authors of the initial assembly acknowledged “an abnormally high percentage of mobile DNA” within the genome in their genome announcement paper, no attempts to retrieve this “mobile DNA” were made (Shapiro et al., 2018) yet.

**TABLE 1:** Putative prophage region predictions in the publicly available complete genome sequences of *Erwinia* strains as identified by PHASTER.

Isolate	Accession number	Length of chromosome (bp)	Total prophage regions/percent of bacterial chromosomes length (%)	Intact	Questionable	Incomplete	Prophage basename
*Erwinia tracheiphila* strain MDcuke	CP013970.1	4891733	28 (17.67%)	19	3	6	ETRACH
*Erwinia billingiae* strain TH88	CP031695.1	4824998	4 (2.13%)	2	0	2	EBILL
*Erwinia persicina* strain B64	CP022725.1	4795673	3 (1.65%)	2	0	1	EPERS
*Erwinia pyrifoliae* DSM 12163	FN392235.1	4026286	6 (4.23%)	1	1	4	EPYRDSM
*Erwinia* sp. QL-Z3	CP037950.1	4926645	4 (2.33%)	1	1	2	ESPQLZ3_
*Erwinia pyrifoliae* strain EpK1/15	CP023567.1	4027225	3 (2.29%)	1	0	2	EPYREPK
*Erwinia* sp. Ejp617	CP002124.1	3909168	3 (1.79%)	0	1	2	N/A
*Erwinia amylovora* strain E-2	CP024970.1	3806898	2 (0.99%)	0	0	2	N/A
*Erwinia amylovora* CFBP1430	FN434113.1	3805573	2 (0.59%)	0	0	2	N/A

### Evolutionary Relationships of Temperate Phages and Prophages of *Erwinia* sp.

**FIGURE 3:**
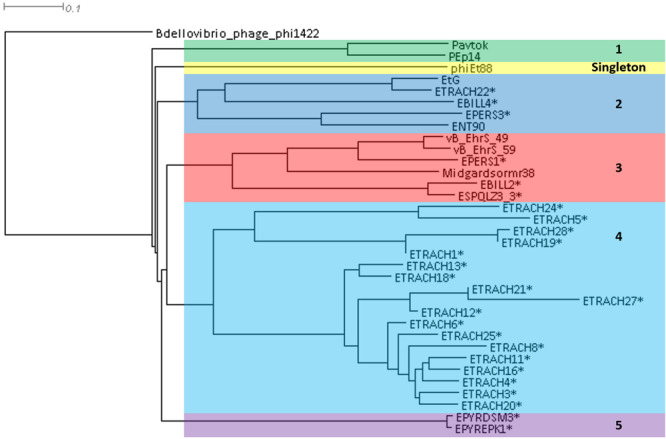
Phylogram based on the complete genome fragmented alignment of temperate *Erwinia* phages and prophages. Cutoff threshold for non-conserved material was set to 5%. *Bdellovibrio* phage phi1422 resembles an outgroup. Colored rectangles indicate similar phage clusters and are numbered. Asterisk indicates prophages derived from *Erwinia* sp. chromosomes. Scale bar represents a 10% difference in average BLASTn score similarity.

A phylogram of the complete genome fragmented alignment of both known temperate *Erwinia* phages and putative prophages suggests at least five distinct clusters of temperate *Erwinia* phages and a singleton—*Erwinia* phage phiEt88 (Figure 3). Basic information on the genomes of temperate *Erwinia* bacteriophages and putative prophages retrieved in this study, as well as proposed further subclustering within clusters 3 and 4, is given in Table 2. Three of the suggested clusters were found to contain only putative prophage regions from *Erwinia* sp. The temperate *Erwinia* phage cluster 1, most evolutionary distant from all the other clades, when all of the putative prophages predicted by PHASTER are retained in the analysis, was found to comprise phages Pavtok and PEp14. Suggested cluster 2 was found to contain both putative prophages from genomes of different *Erwinia* sp. as well as previously described temperate phages EtG and ENT90. Phage EtG seems to be most closely related to one of the putative prophages found in genome of *E. tracheiphila* (ETRACH22), while both of them (EtG and ETRACH22) are also related to one of the putative prophages found in genome of *Erwinia billingiae* (EBILL2). It was noted that three of the aforementioned phages also share similarity with the phage ENT90, which is most closely related to one of the prophages derived from chromosome of *E. persicina* (EPERS3). However, annotation of prophage region number 3 (EPERS3) from *E. persicina* strain B64 revealed it to be a “false positive,” as it lacked most of the core bacteriophage genes and largely represented a tail structural gene module with a few morons, making it a prophage remnant at best; therefore, the decision to exclude it from further analyses was made. Novel *E. persicina* infecting temperate bacteriophage Midgardsormr38 described in this paper was found to cluster together with phages vB_EhrS_49 and vB_EhrS_59, which share a substantial degree of pairwise similarity and were described in detail before by Zlatohurska et al. (2019), along with the putative prophage EPERS1, derived from genome sequence of *E. persicina*, forming subcluster 3.1. Four of the aforementioned phages from subcluster 3.1 were found to be related to the putative prophages derived from the *Erwinia* sp. *QL-Z3* and *E. billingiae* (respectively, ESPQLZ3_3 and EBILL2, which cluster together into subcluster 3.2). The fourth cluster was found to contain 18 putative prophages derived solely from the genome of *E. tracheiphila*. Five prophages of *E. tracheiphila* (ETRACH24, ETRACH5, ETRACH28, ETRACH19, and ETRACH1) formed subcluster 4.1, while 13 remaining *E. tracheiphila* prophages grouped together in a subcluster 4.2, thus making cluster 4 composed exclusively of putative prophages derived from the chromosome of *E. tracheiphila*. Functional annotation of genomes of putative prophages from cluster 4 revealed so many annotation discrepancies and/or lack of identifiable open reading frames encoding essential phage proteins, that cluster 4 was excluded from the majority of further analyses. A high false-positive rate among putative “intact prophage regions” called by the prediction algorithm used was noted at this point. To support this claim, a pairwise genome nucleotide sequence comparison of phages from cluster 4, with predicted functionally grouped ORFs nested, is available in Supplementary Figure S1.

**TABLE 2:** Overview of the analyzed *Erwinia* phages and prophages.

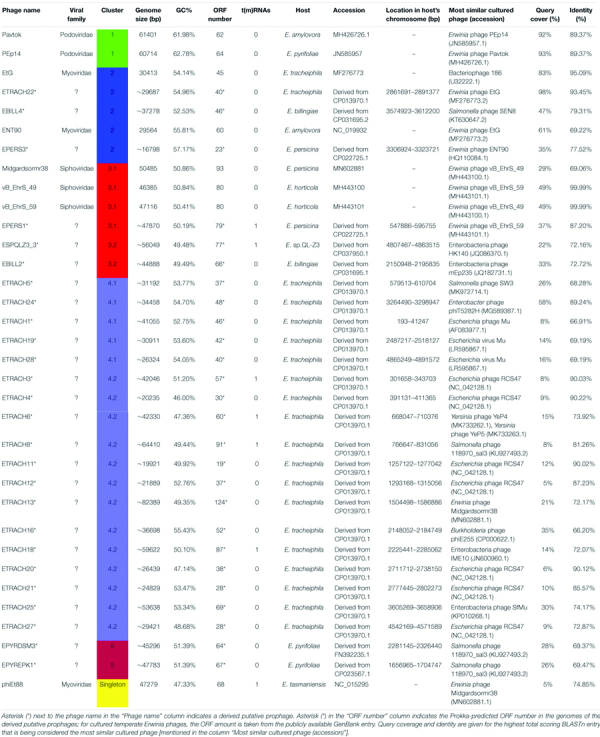

The fifth cluster contained two similar putative prophages derived from two different strains of *Erwinia pyrifoliae*.

Plausibility of the phylogram-suggested clustering of temperate *Erwinia* phages and prophages was confirmed after being manually assessed from a homologous locus group presence and absence table, listing locus groups encoding gene products of at least 50% pairwise identity as inferred by BLASTp (Supplementary Table S2).

The TerL neighbor-joining tree suggests that temperate phages of *Erwinia* sp. mainly employ different types of headful (phages Midgardsormr38, vB_EhrS_49, vB_EhrS_59, Pavtok, Pep14, phiEt88, and prophage EPERS1), 5′ cos (phages ENT90 and EtG and prophages EBILL4, and ETRACH22), and 3′ cos (prophages ESQPLZ3_3, EPYEPK1, EPYRDSM3, and EBILL2) packaging strategies.

**FIGURE 4:**
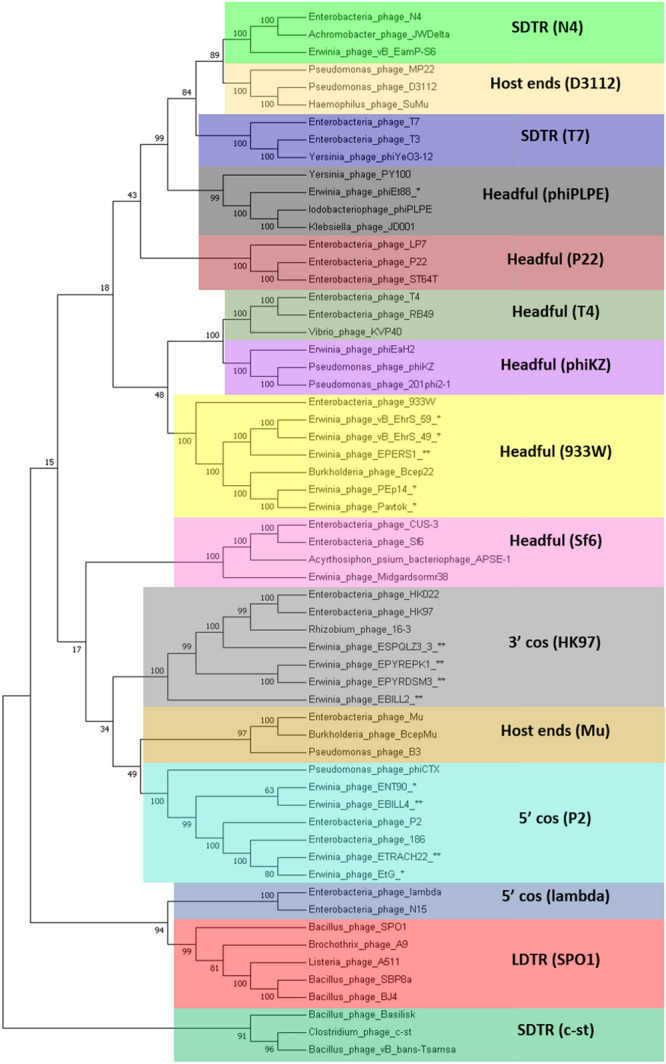
Terminase large subunit protein neighbor-joining tree. The evolutionary history was inferred using the neighbor-joining method (Saitou and Nei, 1987). The bootstrap consensus tree inferred from 1000 replicates is taken to represent the evolutionary history of the taxa analyzed. The percentage of replicate trees in which the associated taxa clustered together in the bootstrap test (1,000 replicates) is shown next to the branches (Felsenstein, 1985). The evolutionary distances were computed using the method of the number of differences (Nei and Kumar, 2000) and are in the units of the number of amino acid differences per sequence. The analysis involved 60 amino acid sequences. All positions with less than 90% site coverage were eliminated. That is, fewer than 10% alignment gaps, missing data, and ambiguous bases were allowed at any position. There were a total of 385 positions in the final dataset. Evolutionary analyses were conducted in MEGA7 (Kumar et al., 2016). Colored rectangles represent groups of phages that employ the same packaging strategy. Asterisk indicates TerL sequence of a known temperate *Erwinia* phage. Double asterisk indicates TerL sequence of a prophage derived from the bacterial chromosome of *Erwinia* sp. LDTR stands for long direct terminal repeats, SDTR for short direct terminal repeats.

Two TerL sequences of phages employing an experimentally confirmed long direct terminal repeat packaging strategy [*Bacillus* phage SBP8a (KX961632.1) and *Bacillus* phage BJ4 (KX961629.1)] were added to the previously mentioned set of sequences for additional LDTR branch support as a high number of phages with unknown packaging strategy (indicated in Figure 4 by asterisk and double asterisk after the phage name) seemed to break the expected prediction tree topology based on individual previously described phage experimental evidence.

### Genomic Comparison of Phages From the Proposed Cluster 1

**FIGURE 5:**
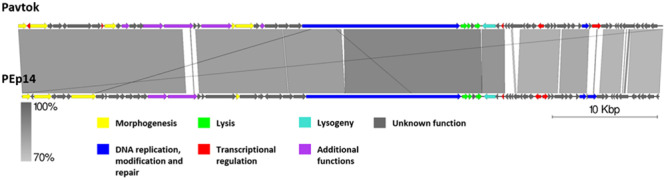
Pairwise genome nucleotide sequence comparison of phages from the suggested cluster 1. Genomes are linearized and drawn to scale; the scale bar indicates 10 thousand base pairs. Arrows representing open reading frames point in the direction of transcription and are color-coded according to the legend. Gray boxes between genomes represent regions of similarity and are gradient-colored according to their identity; darker shade of gray represents higher identity.

Cluster 1 includes phages Pavtok (infecting *E. amylovora* ATCC 29780) and PEp14 (infecting *E. pyrifoliae*), podoviruses of comparable size (∼61 kB), ORF amount, and GC% content of ∼62–63% (Table 2). Despite being isolated on different hosts, these phages seem to be closely related as indicated by average nucleotide identity and general genome collinearity, which was further supported by their shared homologous gene amount (with 53 gene homologs being present in both genomes) (Supplementary Table S3). Lack of nucleotide sequence homology was noted mainly between regions encoding proteins of unknown function located near the genome 3′ termini (Figure 5). Both phages encode EPS depolymerases of near identical size (929 and 930 aa) that share 57.14% amino acid identity over a 99% query coverage. Despite cautious genome functional annotation by authors of both of the submitted genomes, we hypothesize that these EPS depolymerases are virion-associated, rather than soluble enzymes, due to their location within the putative structural gene functional module of these phages (Yurewicz et al., 1971; Knecht et al., 2020). Virion-associated EPS depolymerases play a role in the easening of adsorption to the host; sequence differences of this gene might partially explain the tropism of phages Pavtok and PEp14, since both *E. amylovora* and *E. pyrifoliae* are known to be related, but produce different yet similar in sugar composition and sugar linkages, exopolysaccharides (Kim et al., 2002; Roach et al., 2013). Given that most of the gene products are of comparable size and are homologous to the point that annotation from one of the phages can possibly be extended to the homologous gene of the second phage, annotation of both genomes, thus, could be complemented by such approach. Based on TerL phylogeny, Pavtok and PEp14 both use the headful (933W type) packaging strategy.

### Genomic Comparison of Phages From the Proposed Cluster 2

Cluster 2 comprised two previously described phages, phage EtG (infecting *E. tracheiphila*) and phage ENT90 (infecting *E. amylovora*), and two putative prophages from genomes of *E. tracheiphila* (ETRACH22) and *E. billingiae* (EBILL4). Screening for gene homology revealed 18 gene homologs which are present in genomes of all four phages, majority of them encoding products associated with virion structure and morphogenesis; however, some of them participate in transcriptional regulation, DNA replication, and host lysis (Supplementary Table S3).

**FIGURE 6:**
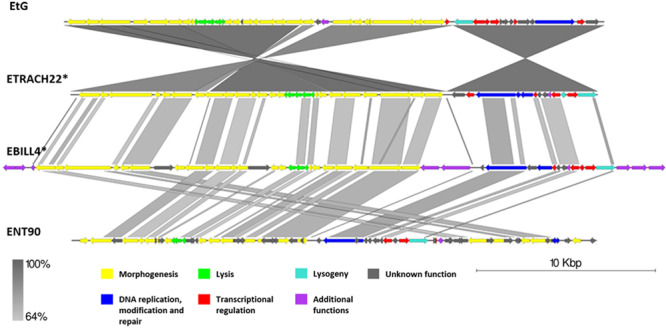
Pairwise genome nucleotide sequence comparison of phages from the suggested cluster 2. Genomes are linearized and drawn to scale; scale bar indicates 10 thousand base pairs. Arrows representing open reading frames point in the direction of transcription and are color-coded according to the legend. Gray boxes between genomes represent regions of similarity and are gradient-colored according to their identity; darker shade of gray represents higher identity. The asterisk next to the phage name indicates a prophage derived from chromosome of *Erwinia* sp.

Phage EtG was found to be most closely related to prophage ETRACH22, as seen by their homologous gene content and pairwise nucleotide sequence similarity. Phage ETRACH22 has 94% query coverage of 95.81% identity to phage EtG, when comparing their genome nucleotide sequences by BLASTn. Both genomes, however, have different genome architectures, showing at least two massive inversion events of different functional modules. It was also noted that phage EtG has insertion of a nucleotide stretch with two ORFs without homology to ORFs in the genome of ETRACH22 in its structural module. Prophage EBILL4 seems to be somewhat collinear to prophage ETRACH22, although having a larger genome and more genes encoding products with functions not necessarily needed for a phage, but possibly beneficial to its host (Supplementary Table S3). As predicted by TerL phylogeny, all four of the phages might employ a 5′ cos packaging strategy. If this is the case, phage ENT90 shows different genomic architectures of ETRACH22 and EBILL4, with genes from structural module located at both 3′ and 5′ termini of its physical DNA molecule (Figure 6).

### Genomic Comparison of Phages From the Proposed Cluster 3

Phages vB_EhrS_49 and vB_EhrS_59 are of similar size and GC% content and share a high degree of similarity in their lysis cassette and part of their structural module, which was previously described in-depth by Zlatohurska et al. (2019). The authors have also noted similarity to one of the prophage regions in the chromosome of *E. persicina* but have not elaborated on it. The sequence of phage EPERS1 was derived from the bacterial chromosome and shows that in its integrated state the genome of EPERS1 does not start from a structural gene module at its 3′ DNA sequence termini. However, as predicted by the TerL phylogeny for phage EPERS1 and noted experimentally for phages Midgardsormr38, vB_EhrS_49, and vB_EhrS_59, phages from subcluster 3.1 employ a headful packaging strategy. As no experimental evidence is yet available on the integration sites of phages Midgadsormr38, vB_EhrS_49, and vB_EhrS_59, circularity of the genomes is assumed due to the headful packaging strategy employed, and as is common in such cases, the terminase small subunit protein (TerS) encoding sequence was chosen to be the first ORF of the non-redundant linearized genomes manually. This suggests that all four of the genomes might be collinear in general and differ mainly in their gene content while retaining the same modular architecture.

**FIGURE 7:**
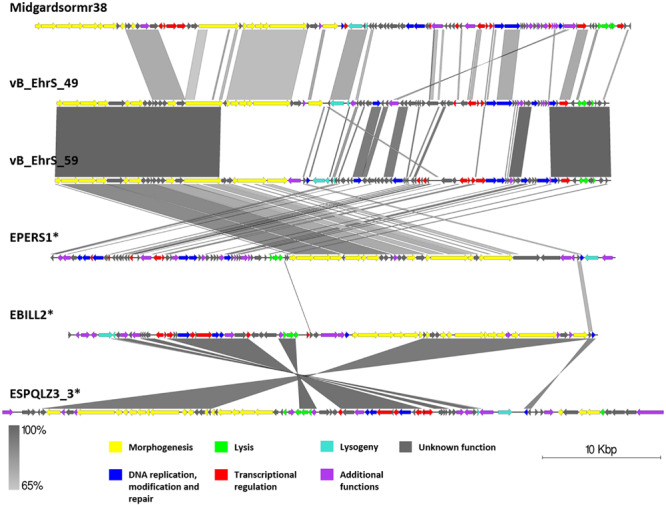
Pairwise genome nucleotide sequence comparison of phages from the suggested cluster 3. Genomes are linearized and drawn to scale; scale bar indicates 10 thousand base pairs. Arrows representing open reading frames point in the direction of transcription and are color-coded according to the legend. Gray boxes between genomes represent regions of similarity and are gradient-colored according to their identity; darker shade of gray represents higher identity. Asterisk next to the phage name indicates a prophage derived from chromosome of *Erwinia* sp.

A pairwise genome nucleotide sequence comparison of prophages ESQPLZ3_3 and EBILL2, interestingly, reveals nucleotide stretches of high homology encompassing ORF encoding proteins hypothetically involved in tail morphogenesis, transcriptional regulation, and lysis, interspaced by stretches of no homology. However, the majority of the ORFs, including the homologous ones, are located on the different DNA strands (Figure 7).

No gene group of 50% pairwise amino acid identity was found to contain genes of all six phages from cluster 3; however, a single hypothetical protein, which seems to be annotated as a DNA polymerase V subunit in various homologous entries, encoding the protein of comparable size, was shared by five of the cluster three phages with the exclusion of vB_EhrS_49. Majority of the gene groups, where the number of isolates ≥2, are made of genes shared by the phages belonging to the same subcluster (either between Midgardsormr38, vB_EhrS_49, vB_EhrS_59, and EPERS1 from subcluster 3.1 or between ESPQLZ3_3 and EBILL2 belonging to subcluster 3.2), although occasional intersubclusteral gene groups do occur (Supplementary Table S3).

The shared gene content analysis also supported the TerL-homology-based packaging inference findings that phages belonging to cluster 3 employ at least three different packaging strategies: Headful (933W type)—vB_EhrS_49, vB_EhrS_59, and prophage EPERS1, 3′ cos—EBILL2 and ESPQLZ3_3, and Headful (Sf6 type)—Midgardsormr38 (Figure 4 and Supplementary Table S3). Although the diversity of headful packaging-type terminases is well acknowledged in the literature and is suggestive of different subtypes of headful packaging, the exact differences which are likely to involve interaction with or recognition of the packaging series initiation site, in the subtypes of headful packaging mechanisms, still remain to be unveiled (Casjens et al., 2005).

### Genomic Comparison of Phages From the Proposed Cluster 4

**FIGURE 8:**
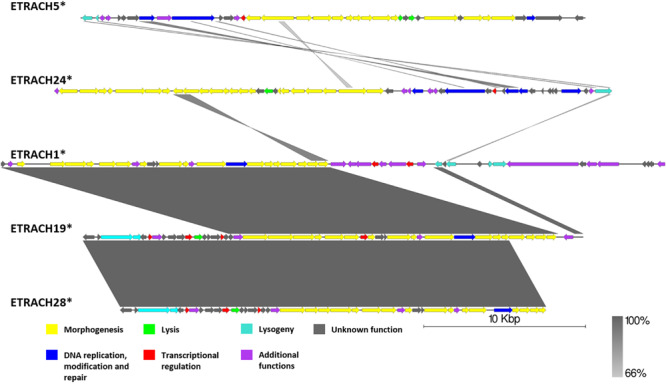
Pairwise genome nucleotide sequence comparison of putative prophage regions from the suggested cluster 4, subcluster 4.1. Genomes are linearized and drawn to scale; scale bar indicates 10 thousand base pairs. Arrows representing open reading frames point in the direction of transcription and are color-coded according to the legend. Gray boxes between genomes represent regions of similarity and are gradient-colored according to their identity; darker shade of gray represents higher identity. The asterisk next to the phage name indicates a prophage derived from chromosome of Erwinia sp.

Although subclustered together on the basis of the average nucleotide identity after fragmented genome alignment and sharing homologous nucleotide stretches, annotation of 13 putative prophage regions from subcluster 4.2 (Supplementary Figure S1) revealed the majority of them to be prophage remnants at best. Not only did these sequences greatly differ by length; most of them lacked genes essential for virion morphogenesis and/or lysis of the host, but contained numerous transposase and transposable element associated genes, revealing defectiveness of these putative prophages on a scale that questions the use of term “prophage” to describe these sequences, despite being regarded as “intact” prophages by PHASTER. Surprisingly, some of the predicted regions from subcluster 4.2 regarded as “intact” lacked any identifiable phage structural genes (e.g., ETRACH27, ETRACH1, and ETRACH20) at all. Predicted prophages from subcluster 4.1 (excluding ETRACH1), on a first glance, seemed more likely truly “intact” than majority of their counterparts from subcluster 4.2 judging by the identifiable core genes in their genome; however, upon closer inspection a question on their completeness persists too (Figure 8 and Supplementary Figure S1). Prophage regions ETRACH1, ETRACH19, and ETRACH28 shared numerous homologous genes from their structural modules, whereas prophage ETRACH28 shares high nucleotide sequence homology with ETRACH19 overall length of its derived genome, with nearly all of the ETRACH28 predicted gene product homologs being present in the genome of ETRACH19. Genomic and genetic proximity of prophages ETRACH19 and ETRACH28 brings up questions on possibility of lysogenization of the same host bacteria by two very closely related temperate phages.

Observations made during analysis of putative prophages regarded as intact from the suggested cluster 4 yet again show us the fact that prophage prediction is indeed a non-trivial task, and, while the scoring systems of prophage prediction algorithms, PHASTER being arguably the most popular one, are being constantly improved, they are still far from the desired positive predictive value of one.

Nevertheless, *E. tracheiphila* strain MDcuke (CP013970.1), which contains up to 28 prophage-like regions which constitute roughly 17.67% of its chromosome length, might be a good example of an object to study the so-called phage domestication phenomenon (Bobay et al., 2014) within the genus *Erwinia*, due to an abnormally high number of cryptic prophages present in the genome of this *E. tracheiphila* strain in comparison with other completely sequenced *Erwinia* sp. (Table 1).

Taken as a whole, “analysis” of controversial cluster 4 highlights the need of “wet-lab” experimental evidence on prophage induction from any of the polylysogens, with *E. tracheiphila* strain MDcuke being an interesting candidate, to unambiguously elucidate intactness of the putative prophages predicted in the complete bacterial genomes *in silico*.

### Genomic Comparison of Phages From the Proposed Cluster 5

**FIGURE 9:**
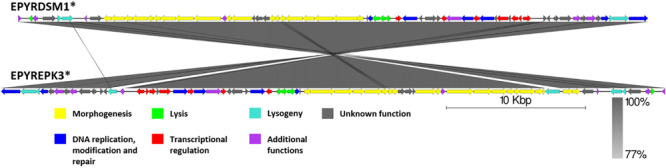
Pairwise genome nucleotide sequence comparison of phages from suggested cluster 5. Genomes are linearized and drawn to scale; scale bar indicates 10 thousand base pairs. Arrows representing open reading frames point in the direction of transcription and are color-coded according to the legend. Gray boxes between genomes represent regions of similarity and are gradient-colored according to their identity; darker shade of gray represents higher identity. Asterisk next to the phage name indicates a prophage derived from chromosome of *Erwinia* sp.

Although, as if integrated on different DNA strands in strains of *E. pyrifoliae*, both EPYRDSM1 and EPYREPK3 seem to be of similar length and contain a comparable amount of open reading frames. The two genomes share 99.87% identity over 99% of the query coverage as compared by BLASTn. Moreover, 63 of the genes were found to be homologous between both prophages. Despite the fact that the genome of EPYREPK3 contains more open reading frames and is slightly longer, these observations suggest a very close evolutionary relationship between these two prophages, suggestive of them being different strains of the same prophage, which have lysogenized different strains of *E. pyrifoliae*. The main differences include two extra regions encoding putative transposases in the genome of EPYREPK3 (Figure 9).

## Conclusion

Fifty-five prophage or prophage-like regions, 26 of which were predicted as “intact” by PHASTER, were found within the 9 publicly available complete genome sequences of *Erwinia* sp. At least five different clusters of temperate *Erwinia* phages sharing a higher degree of similarity were revealed by fragmented complete genome alignments of cultured temperate *Erwinia* phages and the prophages predicted *in silico*, with proposed clustering further supported by their shared homologous gene contents. Manual inspection of the genome annotations for prophages regarded “intact” by the prediction algorithm used revealed a high number of false positives (cryptic prophages or prophage remnants)—regions lacking genes encoding essential phage proteins, among the calls. Packaging strategy experimental evidence or credible prediction has proven itself a necessity, when comparing genome architectures and establishing evolutionary relationships of any given set of related phages and putatively intact prophages. Novel phage Midgardsormr38, the first cultured and sequenced temperate *E. persicina*-infecting phage described herein, is a siphovirus most closely related to the previously cultured *E. horticola*-infecting phages vB_EhrS_49 and vB_EhrS_59 as well as to one of the prophages (EPERS1) predicted in the genome of *E. persicina* B64 (CP022725.1). Each of the *in silico* predicted prophages, regardless of its genome annotation plausibility, however, should be regarded as putative before additional experimental culturing evidence is available.

## Data Availability Statement

The datasets generated for this study are available on request to the corresponding author.

## Author Contributions

AD isolated, propagated, and purified the phage Midgardsormr38 and its host and performed all the microbiological experiments. NZ performed Midgardsormr38 whole-genome sequencing and *de novo* assembly and all the bioinformatic analyses, and wrote the draft version of the manuscript. AK curated the study, analyzed the sequencing data, oversaw the genome functional annotations, and prepared the final version of the manuscript. All authors have conceptualized the study and wrote and edited the manuscript.

## Conflict of Interest

The authors declare that the research was conducted in the absence of any commercial or financial relationships that could be construed as a potential conflict of interest.
