# Supplementary material for: Novel Erwinia persicina Infecting Phage Midgardsormr38 Within the Context of Temperate Erwinia Phages
Source: Front Microbiol. 2020 Jun 19;11:1245. doi: 10.3389/fmicb.2020.01245 (PMC7317114; doi:10.3389/fmicb.2020.01245)
Supplement: Supplementary file 1 [file Image_1.PDF]

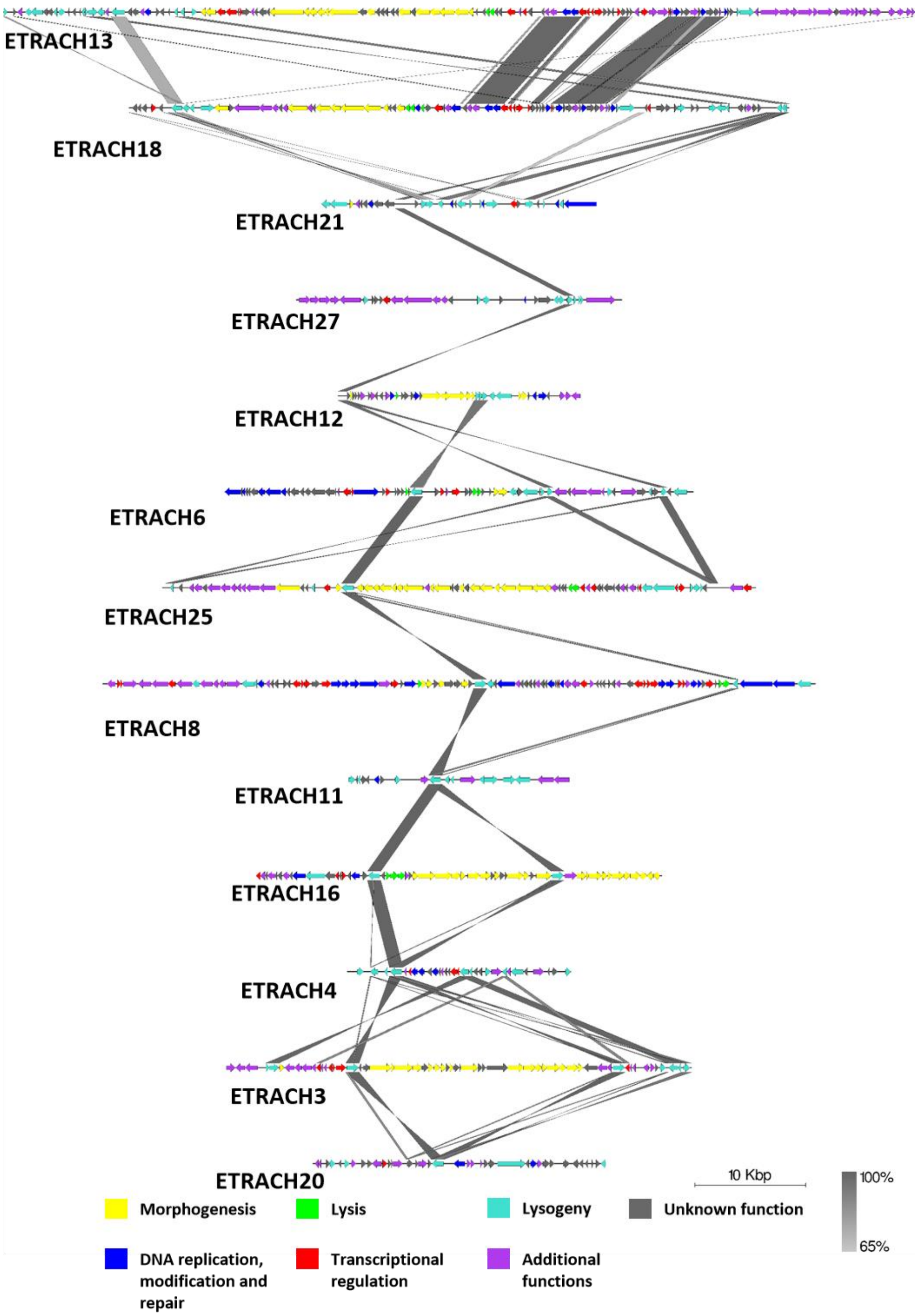

Supplementary Figure 1. Pairwise genome nucleotide sequence comparison of putative prophage regions from the suggested cluster 4, subcluster 4.2. Genomes are linearized and drawn to scale, scale bar indicates 10 thousand base pairs. Arrows representing open reading frames point in the direction of transcription and are color-coded according to the legend. Grey boxes between genomes represent regions of similarity and are gradient-colored according to their identity, darker shade of grey represents higher identity.
