# Supplementary material for: Novel Erwinia persicina Infecting Phage Midgardsormr38 Within the Context of Temperate Erwinia Phages
Source: Front Microbiol. 2020 Jun 19;11:1245. doi: 10.3389/fmicb.2020.01245 (PMC7317114; doi:10.3389/fmicb.2020.01245)
Supplement: Supplementary file 2 [file Data_Sheet_1.PDF]

Supplementary Material

Supplementary Table 1. Homology-based functional annotation of the Midgardsormr38 genome. Homology analysis was performed using BLASTp. Hits with E-values of more than 1E-10 were considered as not significant and are not shown.

Supplementary Table 2. Homologous loci presence/absence table for phages from the suggested clusters 1,2,3 and 5. Columns of the same color represent loci of a phage from a particular cluster and are colored as follows: green – cluster 1, blue – cluster 2, red – cluster 3, purple – cluster 5. Asterisk (*) next to the phage name in the individual phage name column titles indicates that the loci listed in the column are from a putative prophage derived from the complete genome sequence of particular *Erwinia sp*. Note: locus tag numbering by authors of the original complete genome of *Erwinia* phage ENT90 (NC_019932) was not successive in order of appearance and is retained here.

Supplementary Table 3. Excel workbook containing spreadsheets with individual gene presence/absence tables for phages from the suggested clusters 1,2,3 and 5. Asterisk (*) next to the phage name in the individual phage name column titles indicates that the loci listed in the column are from a putative prophage derived from the complete genome sequence of particular *Erwinia sp*. Note: locus tag numbering by authors of the original complete genome of *Erwinia phage* ENT90 (NC_019932) was not successive in order of appearance and is retained here.
